# Supplementary material for: Decline in Other Instrumental Activities of Daily Living as Indicators of Driving Risk in Older Adults at an Academic Memory Clinic
Source: Geriatrics (Basel). 2023 Jan 5;8(1):7. doi: 10.3390/geriatrics8010007 (PMC9844285; doi:10.3390/geriatrics8010007)
Supplement: Supplementary file 1 [file geriatrics-08-00007-s001.zip › geriatrics-2112197-supplementary.pdf]

**Supplementary Materials**

**Supplemental Table S1: Summary of 3MS sub-scores by referral status**

| **3MS Sub-scores** | **Referred drivers** | | **Non-referred drivers** | | **p-value** |
|---|---|---|---|---|---|
| | mean ± SD | N Missing | mean ± SD | N Missing | |
| Birth date and location | 4.8 (0.6) | 0 | 5.0 (0.1) | 0 | 0.12 |
| Registration | 2.8 (0.7) | 0 | 3.0 (0.0) | 0 | 0.05 |
| Mental reversal | 6.0 (1.6) | 0 | 6.7 (1.0) | 0 | 0.01 |
| Immediate recall | 3.9 (2.6) | 0 | 6.1 (2.3) | 0 | <0.05 |
| Temporal orientation | 11.4 (4.7) | 0 | 14.4 (1.9) | 0 | <0.05 |
| Spatial orientation | 4.9 (0.3) | 1 | 5.0 (0.1) | 0 | 0.09 |
| Naming | 4.7 (0.8) | 1 | 4.9 (0.3) | 0 | 0.02 |
| Fluency | 7.1 (2.1) | 0 | 8.6 (1.9) | 0 | <0.05 |
| Abstraction | 3.1 (1.8) | 3 | 4.8 (1.4) | 0 | <0.05 |
| Repetition | 4.8 (0.7) | 1 | 4.9 (0.4) | 0 | 0.35 |
| Read and obey | 2.9 (0.4) | 2 | 3.0 (0.0) | 0 | 0.06 |
| Writing | 4.8 (0.4) | 2 | 5.0 (0.0) | 0 | <0.05 |
| Copying pentagons | 8.7 (1.4) | 2 | 9.4 (0.7) | 0 | <0.05 |
| 3 stage command | 2.8 (0.4) | 6 | 2.9 (0.3) | 0 | 0.45 |
| Delayed recall | 4.8 (2.8) | 0 | 7.5 (2.1) | 0 | <0.05 |

**Supplemental Table S2: Predicted probabilities of being referred to driving authority by number of instrumental activities of daily living (iADL) domains showing decline**

| Clinical Risk factor | Referred drivers | | | | | | | | Non-referred drivers | | | | | | | |
|---|---|---|---|---|---|---|---|---|---|---|---|---|---|---|---|---|
| | N | Mean | SD | Min | Max | 25th Pctl | 50th Pctl | 75th Pctl | N | Mean | SD | Min | Max | 25th Pctl | 50th Pctl | 75th Pctl |
| iADL Functional impairments | | | | | | | | | | | | | | | | |
| 0 to 1 | 18 | 0.37 | 0.2 | 0.1 | 0.6 | 0.2 | 0.4 | 0.5 | 37 | 0.28 | 0.1 | 0.0 | 0.6 | 0.2 | 0.3 | 0.4 |
| 2 | 11 | 0.66 | 0.1 | 0.4 | 0.8 | 0.6 | 0.7 | 0.8 | 10 | 0.55 | 0.2 | 0.1 | 0.8 | 0.4 | 0.6 | 0.7 |
| 3 | 12 | 0.87 | 0.1 | 0.7 | 0.9 | 0.8 | 0.9 | 0.9 | 3 | 0.69 | 0.1 | 0.6 | 0.7 | 0.6 | 0.7 | 0.7 |
| 4 | 9 | 0.86 | 0.1 | 0.7 | 1.0 | 0.8 | 0.9 | 0.9 | | | | | | | | |

**Supplement Table S3: Baseline characteristics by referral status and number of instrumental activities of daily living (iADL) domains showing decline**

| | **Referred drivers (N=50)** | | **Non-referred drivers (N=50)** | |
|---|---|---|---|---|
| | *Number of iADL impairments* | | *Number of iADL impairments* | |
| | 0 to 1 (N=18) | 2 to 4 (N=32) | 0 to 1 (N=37) | 2 to 4 (N=13) |
| Age (years), mean ± SD | 76.8 ± 8.4 | 78.9 ± 5.3 | 72.9 ± 7.0 | 72.9 ± 7.0 |
| Sex, n (%) | | | | |
| Male | 15 (83.3) | 16 (50.0) | 16 (43.2) | 9 (69.2) |
| Female | 3 (16.7) | 16 (50.0) | 21 (56.8) | 4 (30.8) |
| Education, n (%) | | | | |
| High school or lower | 6 (33.3) | 17 (53.1) | 11 (29.7) | 3 (23.1) |
| College or university | 12 (66.7) | 15 (46.9) | 26 (70.3) | 10 (76.9) |
| First language, n (%) | | | | |
| English | 8 (44.4) | 17 (53.1) | 13 (35.1) | 4 (30.8) |
| French | 10 (55.6) | 15 (46.9) | 22 (59.5) | 8 (61.5) |
| Other | 0 (0.0) | 0 (0.0) | 2 (5.4) | 1 (7.7) |
| Living status, n (%) | | | | |
| With others | 15 (83.3) | 24 (75.0) | 32 (86.5) | 12 (92.3) |
| Alone | 3 (16.7) | 8 (25.0) | 5 (13.5) | 1 (7.7) |
| Cardiovascular disease, n (%) | | | | |
| Yes | 15 (83.3) | 23 (71.9) | 28 (75.7) | 13 (100) |
| No | 3 (16.7) | 9 (28.1) | 9 (24.3) | 0 (0.0) |
| Psychiatric disease, n (%) | | | | |
| Yes | 7 (38.9) | 11 (34.4) | 12 (32.4) | 5 (38.5) |
| No | 11 (61.1) | 21 (65.6) | 25 (67.6) | 8 (61.5) |
| Number of medications, mean ± SD | 4.2 ± 2.9 | 4.4 ± 2.7 | 3.9 ± 2.5 | 4.8 ± 1.7 |

**Supplement Table S4: Description of clinical risk factors for driving by referral status and number of instrumental activities of daily living (iADL) domains showing decline**

| | **Referred drivers (N=50)** | | | **Non-referred drivers (N=50)** | | |
|---|---|---|---|---|---|---|
| | *Number of iADL impairments* | | | *Number of iADL impairments* | | |
| | 0 to 1 (N=18) | 2 to 4 (N=32) | N Missing | 0 to 1 (N=37) | 2 to 4 (N=13) | N Missing |
| Trails B test (seconds), mean ± SD | 263.3 ± 54.7 | 219.8 ± 85.2 | 3 | 125.8 ± 49.0 | 102.7 ± 27.7 | 1 |
| Trails B score categories, n (%) | | | | | | |
| Below 3 minutes | 3 (16.7) | 11 (37.9) | 3 | 33 (91.7) | 13 (100) | 1 |
| 3 minutes or above | 15 (83.3) | 18 (62.1) | | 3 (8.3) | 0 (0.0) | |
| 3MS Total score, mean ± SD | 83.7 ± 9.6 | 71.6 ± 16.7 | 0 | 91.8 ± 7.1 | 89.4 ± 9.3 | 0 |
| Clock drawing score categories, n (%) | | | | | | |
| Score 1-2 | 11 (61.1) | 15 (48.4) | 1 | 35 (94.6) | 11 (84.6) | 0 |
| Score 3-4 | 7 (38.9) | 16 (51.6) | | 2 (5.4) | 2 (15.4) | |
